# Regulation of downstream neuronal genes by proneural transcription factors during initial neurogenesis in the vertebrate brain

**DOI:** 10.1186/s13064-016-0077-7

**Published:** 2016-12-07

**Authors:** Michelle Ware, Houda Hamdi-Rozé, Julien Le Friec, Véronique David, Valérie Dupé

**Affiliations:** 1 Institut de Génétique et Développement de Rennes, Faculté de Médecine, CNRS UMR6290, Université de Rennes 1, IFR140 GFAS, 2 Avenue du Pr. Léon Bernard, 35043 Rennes Cedex, France; 2 Laboratoire de Génétique Moléculaire, CHU Pontchaillou, Rennes Cedex, France; 3 Present address: Department of Physiology, Development and Neuroscience, University of Cambridge, Anatomy Building, Downing Street, CB2 3DY Cambridge, UK

**Keywords:** Notch, Embryonic, Early axon scaffold, Neurogenin, Ascl1, Rbpj, Tagln3, Chga

## Abstract

**Background:**

Neurons arise in very specific regions of the neural tube, controlled by components of the Notch signalling pathway, proneural genes, and other bHLH transcription factors. How these specific neuronal areas in the brain are generated during development is just beginning to be elucidated. Notably, the critical role of proneural genes during differentiation of the neuronal populations that give rise to the early axon scaffold in the developing brain is not understood. The regulation of their downstream effectors remains poorly defined.

**Results:**

This study provides the first overview of the spatiotemporal expression of proneural genes in the neuronal populations of the early axon scaffold in both chick and mouse. Overexpression studies and mutant mice have identified a number of specific neuronal genes that are targets of proneural transcription factors in these neuronal populations.

**Conclusion:**

Together, these results improve our understanding of the molecular mechanisms involved in differentiation of the first neuronal populations in the brain.

**Electronic supplementary material:**

The online version of this article (doi:10.1186/s13064-016-0077-7) contains supplementary material, which is available to authorized users.

## Background

In the embryonic rostral brain, the first neurons differentiate in very specific domains and project axons to give rise to the early axon scaffold. This is an evolutionary conserved structure, formed from longitudinal, transversal and commissural axon tracts that act as a scaffold for the guidance of later axons [12, 55, 57, 59]. Each tract is formed from a small neuronal population, including the nucleus of the medial longitudinal fascicle (nMLF), the nucleus of the tract of the postoptic commissure (nTPOC), the nucleus of the mammillotegmental tract (nMTT), the nucleus of the tract of the posterior commissure (nTPC) and the nucleus of the descending tract of the mesencephalic nucleus of the trigeminal nerve (nmesV) (see Table 1 for abbreviations). Despite the importance of these tracts for ensuring the correct formation of later complex connections, the molecular mechanisms involved in differentiation and specification of the neuronal populations that give rise to the early axon scaffold tracts has largely been ignored.

**Table 1:** Abbreviations used throughout the paper

Cda	Circumferential descending axons
Di	diencephalon
dCortex	dorsal cortex
DMB	diencephalic-mesencephalic boundary
DTmesV	descending tract of the mesencephalic nucleus of the trigeminal nerve
Ep	epiphysis
LC	locus coeruleus
Mes	mesencephalon
MLF	medial longitudinal fascicle
MRB	mesencephalic-rhobencephalic boundary
MTT	mammilotegmental tract
nIII	nucleus of the oculomotor nerve
nIV	nucleus of the trochlear nerve
nmesV	nucleus of the descending tract of the mesencephalic nucleus of the trigeminal nerve
nMLF	nucleus of the medial longitudinal fascicle
nMTT	nucleus of the tract of the mammilotegmental tract
nTPC	nucleus of the tract of the posterior commissure
nTPOC	nucleus of the tract of the postoptic commissure
Os	optic stalk
p1, p2, p3	prosomere 1, prosomere 2, prosomere 3
pros	prosencephalon
Ptec	pretectum
Pth	prethalamus
Rh	rhombencephalon
Tel	telencephalon
TPC	tract of the posterior commissure
TPOC	tract of the postoptic commissure
vCortex	ventral cortex

In all neuronal tissue, expression of specific neuronal transcription factors needs to be tightly controlled to ensure the correct patterning of neuronal populations both temporally and spatially [3]. This patterning is regulated in part by the Notch signalling pathway, which has remained highly conserved throughout vertebrate evolution. Lateral inhibition with feedback regulation allows Notch signalling to maintain the number of neural progenitor cells (NPCs) by controlling the number of neighbouring cells that can exit the cell cycle and subsequently undergo neural differentiation [14]. Cell cycle exit is controlled by a limited number of basic helix-loop-helix (bHLH) proneural genes that are both necessary and sufficient to activate neurogenesis [5, 28]. Loss of function studies indicate that proneural transcription factors direct not only general aspects of neuronal differentiation, but also specific aspects of neuronal identity within NPCs [23, 39, 60]. These proneural transcription factors include ASCL1 and members of the Neurogenin family. In many neuronal tissues these proneural genes are expressed in complementary domains [5, 13, 32, 37], suggesting that they contribute to the specificity of neuronal populations. In recent years, there has been emphasis on determining their downstream target genes, with proneural transcription factors playing a pivotal role in the transcriptional cascade that specifies neurons by activating general neuronal markers, either directly or indirectly [21]. Global profiling approaches are beginning to identify a large number of target genes that could be directly regulated by ASCL1 [2, 8, 16, 50, 58]. Recently, by inhibiting the Notch signalling pathway with the chemical inhibitor N-[3.5-difluorophenacetyl-L-alanyl)]-S-phenylglycine t-butyl ester (DAPT) during early chick development, new neuronal markers including Transgelin 3 (*Tagln3*), Chromogranin A (*Chga*) and Contactin 2 (*Cntn2*) were identified and introduced to a network of downstream proneural targets genes [43]. Analysis of their expression, as well as the known neuronal markers, *Nhlh1* and Stathmin 2 (*Stmn2*), revealed interesting patterns overlapping with the first neuronal populations of the early axon scaffold in the developing chick brain [44].

Identifying gene regulatory networks are essential for understanding the molecular cascades involved in subtype specification of neurons. Here, we describe the molecular cascade implicating Notch signalling, proneural genes and downstream targets at the level of the first neuronal populations that give rise to the early axon scaffold in both chick and mouse embryos. We identified several target genes that are known neuronal markers (*Nhlh1*, *Tagln3*, *Chga, Cntn2* and *Stmn2*), which are likely to play an essential role in the differentiation of these neuronal populations.

## Methods

### Chick embryos

Fertilised chicken (*Gallus gallus*) eggs were obtained from E.A.R.L. Les Bruyères (France). Eggs were incubated in a humidified incubator at 38 °C until the required developmental stages described according to Hamburger and Hamilton [19].

### Generation and genotyping of mutant mouse embryos

To generate conditional RBPj knock-out mice, RBPJ^f/f^ [20] mice were crossed with R26R^creERT2^ [3] mice. To activate cre recombinase, tamoxifen (Sigma) was dissolved in sunflower oil at a concentration of 10 mg/ml. 5 mg of tamoxifen was injected by intraperitoneal (IP) injection at embryonic day (E) 7.5 and embryos were harvested at E9.5. Heterozygous Ascl1 delta null mutant mice were used in this study [18]. Genotyping of RBPj mutant embryos and Ascl1 delta null mutant embryos was performed as previously described [7, 20]. Animal experimentation protocols were reviewed and approved by the Direction Départementale des Services Vétérinaires and are conformed to the European Union guidelines (RL2010/63/EU).

### *In ovo* electroporation

The pCAGGS-IRES-nuclearGFP (pCIG) plasmid was used for control experiments. The overexpression constructs for rat *Ascl1* and mouse *Neurog2* were previously cloned into the pCIG plasmid [9]. The expression constructs were used at a concentration of 1 μg/μL^−1^, with Fast Green (Sigma) added at 0.2% to facilitate visualisation of the DNA solution. The DNA solution was injected into the rostral neural tube of chick embryos at Hamburger and Hamilton stage (HH) 10-11, using a nanoinjector (Drummond Scientific). Electrodes were placed either side of the neural tube, targeting the mesencephalon. Five pulses of 15 V/50 ms were applied, using a square wave pulse electroporator (CUY21SC; Nepa Gene Co., Ltd). After electroporation, the eggs were sealed and incubated for a further 24 h.

### *In situ* hybridisation and immunohistochemistry

All embryos were fixed in 4% PFA/PBS at 4 °C overnight, rinsed and processed for whole-mount RNA *in situ* hybridisation or immunohistochemistry. Anti-sense probes were generated either from plasmids cloned as previously described [43] or plasmids provided as a gift. The protocol for single and double *in situ* hybridisation has been previously described [43]. For double labelling, Digoxigenin and Fluorescein labelled probes were incubated together. The Digoxigenin antibody (Roche) was added first, followed by the NBT/BCIP reaction. After inactivation of the colour reaction, the embryos were fixed with 4% PFA overnight, then the Fluorescein antibody (Roche) was added, followed by fast red reaction (VectorRed). The immunohistochemistry protocol with anti-HuC/D (1:500; molecular probes; A21271) and anti-neurofilament (1:1000; Invitrogen; 13–0700) has previously been described [30].

## Results

### Expression of neuronal markers during early development of the mouse brain

**Fig. 1:**
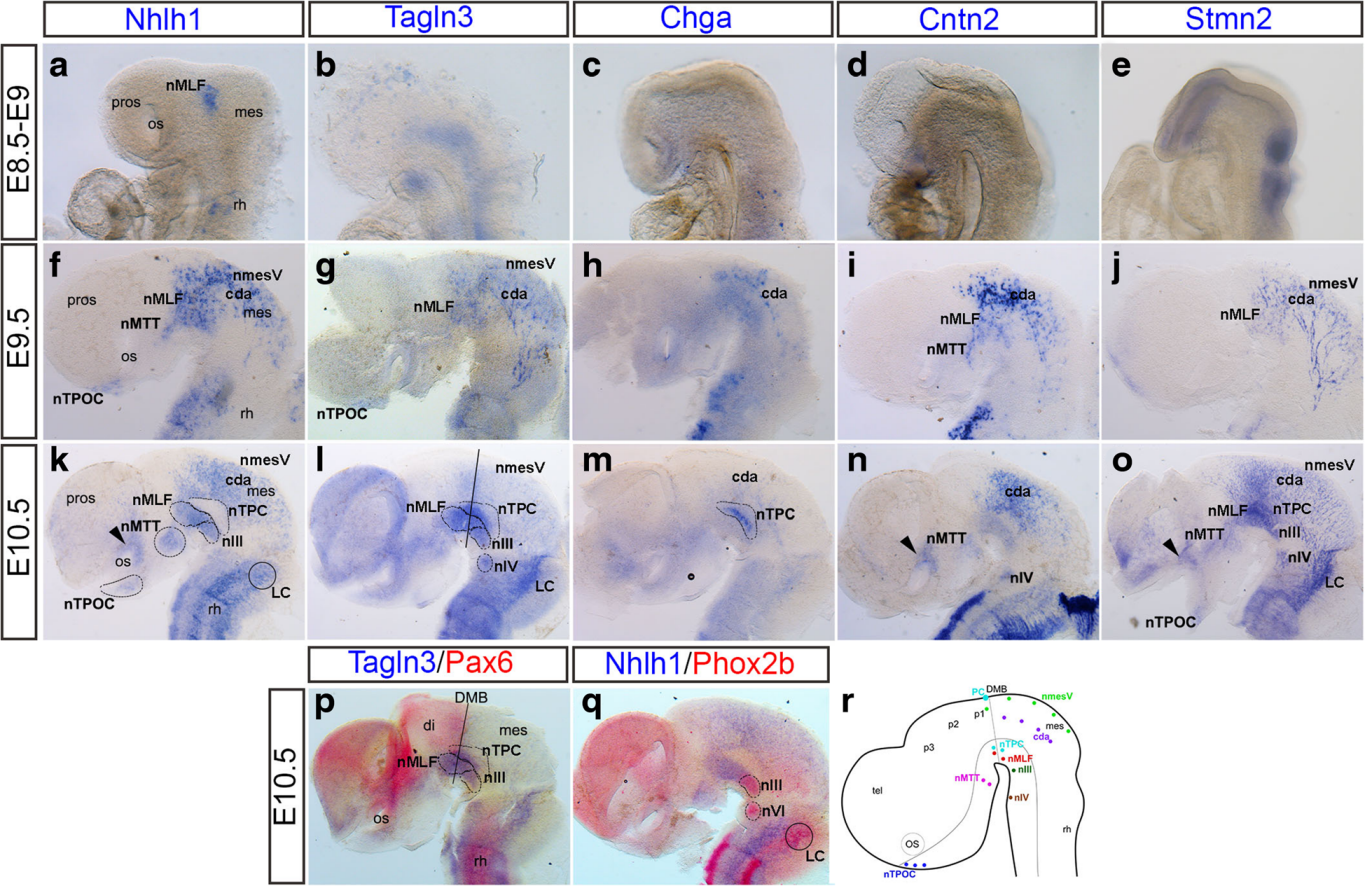
Expression of neuronal markers between E8.5 and E10.5 in the developing mouse brain. All brains have been dissected and flatmounted in lateral view. **a** E9, *Nhlh1* expression in the ventral midline corresponding to the nMLF. **b** E8.5, *Tagln3* expression was ubiquitous through the ventral midline. **c**, **d** E8.5, *Chga and Cntn2,* no expression in the brain. **e** E8.5, *Stmn2* expression in the rhombencephalon and rostral neural folds. At E9.5, expression of *Nhlh1* (**f**), *Tagln3* (**g**), *Chga* (**h**), *Cntn2* (**i**) and *Stmn2* (**j**) was present throughout the neuronal populations of the early axon scaffold tracts. At E10.5, expression of *Nhlh1* (**k**), *Tagln3* (**l**), *Chga* (**m**), *Cntn2* (**n**) and *Stmn2* (**o**) in neuronal populations of the established early axon scaffold (as delimited by dashed lined areas in **k** and **l**). There was also expression in the motor neurons, nIII and nIV. Arrowhead indicated expression of *Nhlh1*, *Cntn2* and *Stmn2* in the optic vesicle. In the rhombencephalon there was expression throughout the rhomomeres and locus coeruleus (LC). **p** E10.5, location of DMB (black longitudinal line) revealed by *Pax6* in relation to *Tagln3* expression. **q** E10.5, location of the nIII and nIV as well as the LC revealed by *Phox2b* compared with *Nhlh1*. **r** Schematic of early axon scaffold neuronal populations in the rostral brain. Each population has been colour coded. Grey longitudinal line represented the alar-basal boundary. Grey transversal line represented the DMB. For abbreviations see Table 1

Recently, a number of neuronal markers, described as part of the Notch/proneural network, were shown to be specifically expressed in the early neuronal populations of the chick brain [44]. To investigate the role of this network during formation of these neuronal populations in the developing mouse brain, the expression patterns of those markers, *Nhlh1*, *Tagln3*, *Chga*, *Cntn2* and *Stmn2* were analysed between E8.5 and E10.5 (Fig. 1). The conservation of gene expression was analysed by comparison with chick data (Table 2). Similar to the expression patterns observed in the chick embryo [44], these neuronal markers were differentially expressed throughout the early neuronal populations in the brain (Fig. 1 and Table 2), cranial ganglia and spinal cord (data not shown) in the developing mouse embryo. We show that these genes were not pan-neuronal markers, but instead have characteristic expression domains at the level of these first neuronal populations developing in the brain.

**Table 2:** Expression of *Nhlh1*, *Tagln3*, *Chga*, *Cntn2* and *Stmn2* in the developing chick and mouse brains

	Mouse E9.5-E10.5					Chick HH12-HH17				
	*Nhlh1*	*Tagln3*	*Chga*	*Cntn2*	*Stmn2*	*Nhlh1*	*Tagln3*	*Chga*	*Cntn2*	*Stmn2*
cda	✓	✓	✓	✓	✓	N/A	N/A	N/A	N/A	N/A
nmesV	✓	✓			✓	✓				✓
nMLF	✓	✓		✓	✓	✓	✓			✓
nMTT	✓	✓		✓	✓	✓	✓		✓	✓
nTPC		✓	✓		✓			✓		
nTPOC	✓	✓			✓	✓	✓	✓		✓
nIII	✓	✓			✓	✓	✓		✓	✓
nIV		✓		✓	✓	✓	✓		✓	✓

Ticks indicate where expression was present in the early axon scaffold populations and the motor neurons. Expression in the mouse brain between E9.5 and E10.5, compared in the chick brain between HH12 and HH17 (taken from [44] and Fig. 7)

At E8.5, there was no expression of these markers along the dorsal midline corresponding to the nmesV (Fig. 1a-e). This was surprising as the nmesV were the first neurons to arise in the rostral brain at E8.5 [12] and expression of *Nhlh1* and *Tagln3* predated the appearance of neurons in the chick brain [44]. *Nhlh1* expression was the first of these markers to be switched on in the ventral diencephalon corresponding to the nMLF (Fig. 1a). *Tagln3* was ubiquitously expressed throughout the ventral brain (Fig. 1b), while *Chga* and *Cntn2* were not yet expressed (Fig. 1c, d). *Stmn2* was expressed at E8.5 in the rostral prosencephalon and the rhombencephalon (Fig. 1e). At E9.5, expression of these markers were switched on in various neuronal populations (Fig. 1f-j and Table 2).

By E10.5, *Nhlh1*, *Tagln3* and *Stmn2* were expressed in almost all the neuronal populations of the brain (Fig. 1 k, l, o), while *Chga* and *Cntn2* were expressed more specifically (Fig. 1m, n). There was a clear gap between the circumferential descending axons (cda) and the nMLF where *Nhlh1* and *Cntn2* were not expressed (Fig. 1k, n), correlating to where the nTPC neurons were located. In contract, *Tagln3*, *Chga* and *Stmn2* were expressed in the nTPC (Fig. 1l, m, o). Double labelling with *Pax6* (Fig. 1p) was used to mark the diencephalic-mesencephalic boundary (DMB) and confirmed the expression of *Tagln3* in the nMLF and nTPC within both the diencephalon and mesencephalon [33].

During development of the early axon scaffold, the oculomotor (III) and trochlear (IV) motor neurons also differentiated at the ventral midline. As the nucleus of the oculomotor nerve (nIII) was not easily identifiable from the nMLF and nTPC at E10.5. Therefore, *Phox2b* was used as a specific marker of the motor neurons [40] to distinguish these populations (Fig. 1q). All the neuronal markers except *Chga* were expressed in the nIII (Fig. 1k-o). *Tagln3*, *Cntn2* and *Stmn2* were expressed in the nucleus of the trochlear nerve (nIV) (Fig. 1l, n, o).

While the expression of these markers in the mouse brain was largely conserved with chick, there were some subtle differences. For example, *Chga* was not expressed along the dorsal midline of the mesencephalon in the mouse (Fig. 1h, m and Table 2). Similar to chick, expression of *Cntn2* was not expressed in the nmesV along the mesencephalic roof, but in contract *Cntn2* was expressed in the cda neurons in the mouse mesencephalon (Fig. 1i, n). Expression of the later markers, *Chga*, *Cntn2* and *Stmn2* in the mesencephalon at E9.5 suggested cda neurons were already present at this stage (Fig. 1h, i, j). The cda neurons were likely to be homologous to the tectobulbar neurons in the chick brain [27]. However, there was no expression of these neuronal markers in the same region of the chick mesencephalon suggesting differences in neuronal differentiation of these neurons (Table 2).

Having described the expression of these genes within the early neuronal populations in the mouse brain (Fig. 1r), the goal of this study was to determine what regulated the expression of these genes during initial neurogenesis in the rostral brain and during early axon scaffold formation. Having previously shown the involvement of the Notch signalling pathway in the expression of *Nhlh1*, *Tagln3*, *Chga*, *Cntn2* and *Stmn2* in chick, we first looked at the Notch/proneural network [43].

### Expression of *Ascl1* and neuronal markers in the early neuronal populations in the brain was regulated by Notch signalling in mouse

**Fig. 2:**
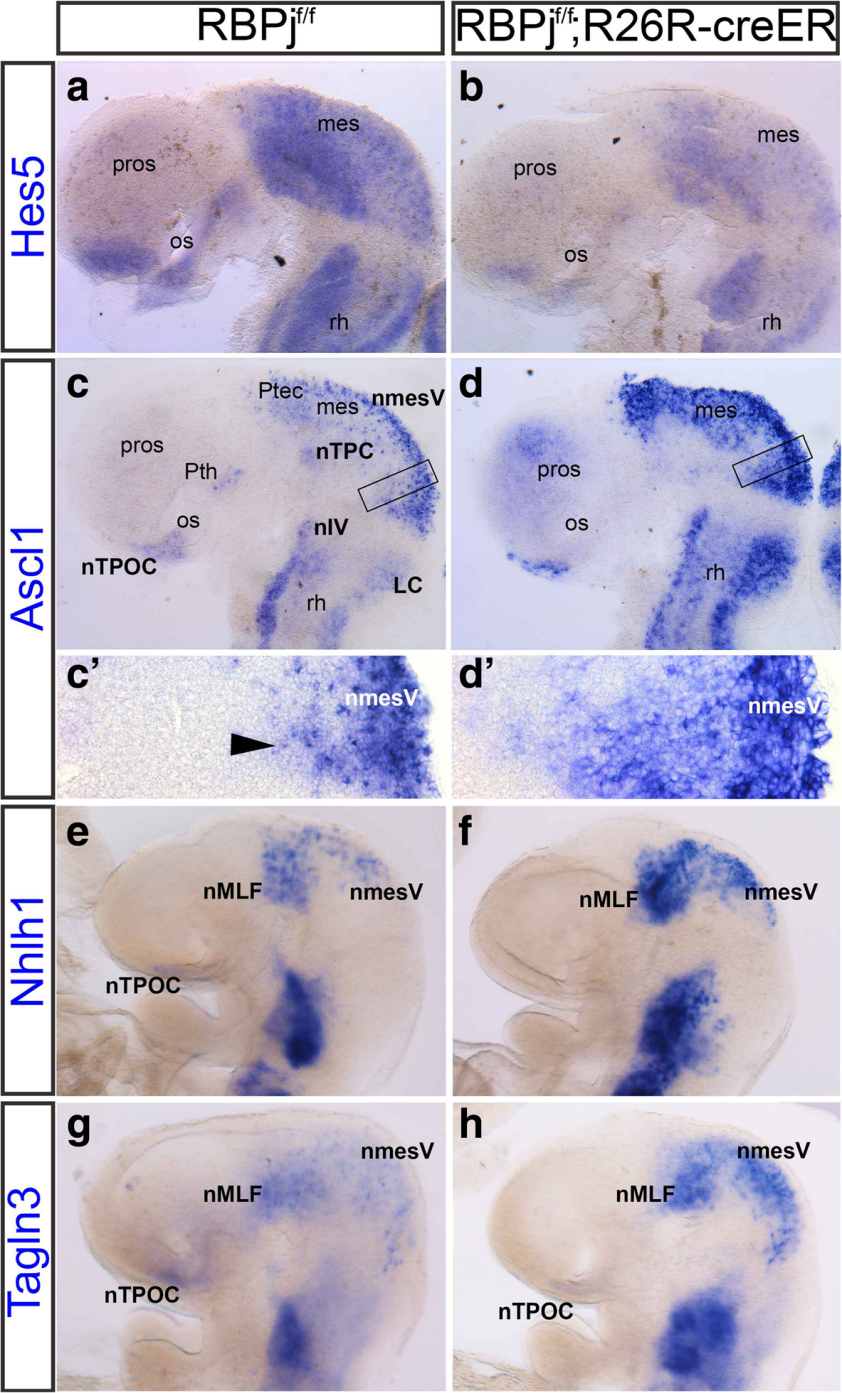
Loss of Notch signalling affects expression of *Hes5, Ascl1*, *Nhlh1* and *Tagln3* in the mouse brain. (**a**-**d**) All brains have been dissected and flatmounted in lateral view. **e**-**h** Whole mount embryos. **a**, **b**, *n* = 10 Expression of *Hes5* at E9.5 within the embryonic mouse brain of the control (**a**) and RBPJ mutant (**b**). **c**, *c’*, **d**, *d’*, *n* = 10 *Ascl1* expression in the neuronal populations, which give rise to the early axon scaffold tracts at E9.5 of the control (**c**, *c’*) and RBPj mutant brains (**d**, *d’*). Boxes in **c** and **d** indicate higher magnification in *c’* and *d’* respectively. Arrowhead indicates normal salt-and-pepper like expression of *Ascl1*. Control and mutant embryos were compared from the same littermates. **e**, **f**, *n* = 5 *Nhlh1* expression in control (**e**) and RBPj mutant (**f**). **g**, **h**, *n* = 5 *Tagln3* expression in control (**g**) and RBPj mutant (**h**). Expression of *Nhlh1* and *Tagln3* was upregulated throughout the brain. For abbreviations see Table 1

So far, *Ascl1* has been the only proneural gene to have its expression described in detail during formation of the early neuronal populations in the mouse brain. Expression was first detected in the brain at E8.0 in the nmesV before neuronal differentiation [34, 56]. We wanted to determine if the relationship between *Ascl1* and Notch signalling was similar to that already described in other central nervous system regions [47]. RBPj mutant mice have been commonly used to study the role of Notch inhibition [11, 36]. However, as the full RBPj knock-out mouse was embryonic lethal at E9, before the neuronal populations of the early axon scaffold tracts were fully established, we created a conditional mutant mouse by crossing RBPj^f/f^ [20] and R26R^creERT2^ mice [3]. Initially pregnant females were injected with 5 mg of tamoxifen at E6.5, before Notch signalling was active in the brain. However, the embryos displayed a typical Notch deficient phenotype with a strong developmental delay and it was not possible to compare brain development from this stage (results not shown). After injection of 5 mg tamoxifen, one day later at E7.5, we were able to rescue the early lethality and obtained RBPj^f/f^;R26R^creERT2^ embryos with an apparent similar morphology to the control embryos at E9.5. To confirm Notch signalling was knocked down in these embryos, *Hes5* expression was analysed (Fig. 2a, b; *n* = 10). *Hes5* was downregulated, but expression was not completely lost throughout the RBPj mutant brain (Fig. 2b). This result indicated a partial inhibition of Notch was established in these RBPj mutant embryos.

In the control embryos, *Ascl1* was normally expressed throughout the early neuronal populations, including the nTPOC, nmesV and nTPC (Fig. 2c, c'; *n* = 10). There was also expression along the dorsal and ventral rhombencephalon, the locus coeruleus (LC), the pretectum (Ptec) and the prethalamus (Pth) (Fig. 2c). Expression in the control brain was in a salt-and-pepper like pattern (Fig. 2c’, arrowhead). When Notch signalling was knocked down, *Ascl1* expression was upregulated throughout the RBPj mutant brain and the salt-and-pepper like pattern was lost (Fig. 2d, d’; *n* = 10). Although *Ascl1* expression was upregulated, the neuronal populations remained identifiable. This showed that Notch signalling negatively regulates neurogenesis and that lateral inhibition involving *Ascl1* was implicated in the differentiation of the neuronal populations of the early axon scaffold tracts in mouse brain.

Compared to control embryos, there was no *Ascl1* expression in some regions of these RBPj mutant brains, such as, the Pth and nTPC. As *Ascl1* should be expressed in these populations already, this suggested there was already a developmental delay in these mutant embryos (Fig. 2d).

Using this RBPj mutant model, we also investigated the expression of the pan-neuronal markers, *Nhlh1* and *Tagln3* (Fig. 2e-h; *n* = 5). Both genes were upregulated throughout the neuronal populations that give rise to the early axon scaffold tracts, which genetically confirmed expression of these genes was regulated by the Notch pathway (Fig. 2f, h).

### Complementary and restricted expression of proneural genes in the developing mouse brain

**Fig. 3:**
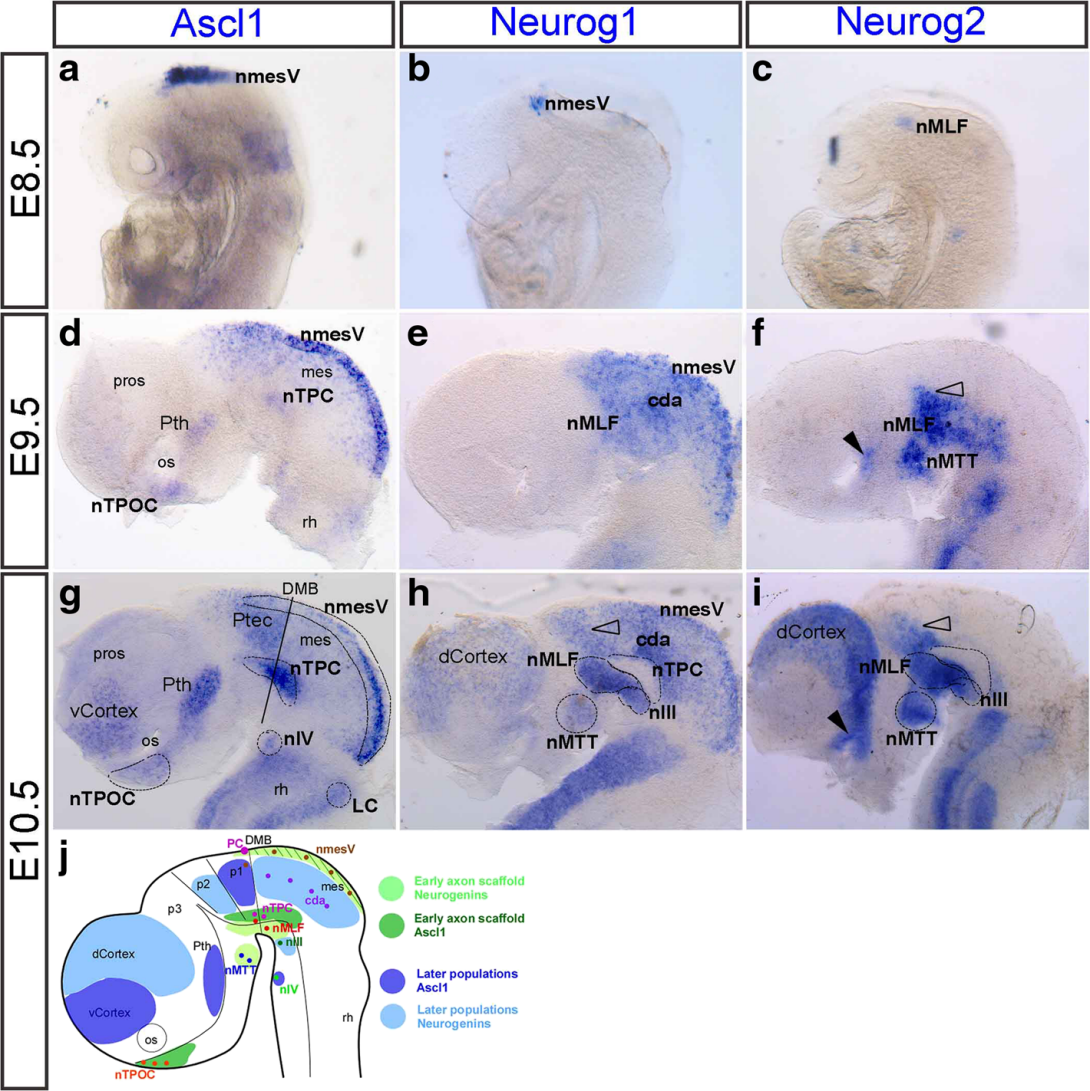
Expression of proneural genes in the mouse brain from E8.5-E10.5. **a**-**c** E8.5 (lateral views), expression of *Ascl1* (**a**) and *Neurog1* (**b**) along the dorsal midline of the mesencephalon corresponding to the nmesV. Expression of *Neurog2* (**c**) in the ventral brain, corresponding to the nMLF. **d**-**i** All brains have been dissected, flatmounted and in lateral view. **d**-**f** E9.5, expression of *Ascl1* (**d**), *Neurog1* (**e**) and *Neurog2* (**f**). **f** Arrowhead indicates expression in the dorsal optic vesicle. **g**-**i** E10.5, expression of *Ascl1* (**g**), *Neurog1* (**h**) and *Neurog2* (**i**) within the neuronal populations of the early axon scaffold tracts and motor neurons as delimited by dashed lines. Unfilled arrowhead indicated caudal thalamus. There were other areas of the brain that expressed *Ascl1*, including the ventral cortex, pretectum and prethalamus. *Neurog1* and *Neurog2* were both expressed in the dorsal cortex, the dorsal optic vesicle (arrowhead) and the caudal thalamus (unfilled arrowhead). **j** Schematic of neuronal populations and complementary expression in these early neuronal populations of *Ascl1* (dark green) and neurogenins (light green) and in other regions *Ascl1* (dark blue) and Neurogenins (light blue). For abbreviations see Table 1

As proneural genes are essential transcription factors for neurogenesis [5], we wanted to determine whether they played a role in regulating the expression of these neuronal markers. While the expression patterns of proneural genes have been widely described in populations throughout the peripheral and central nervous systems [18, 31, 32, 48], a detailed description during initial neurogenesis in the brain was lacking. Therefore, we first needed to confirm the expression patterns of proneural genes in these early neuronal populations. The expression patterns of *Neurog1* and *Neurog2* were analysed in the developing mouse brain in comparison to *Ascl1* (Fig. 3 and Table 3). Other proneural genes were not described here, such as *Atoh1*, which was not expressed in the ventral brain (data not shown) and *Neurog3* was only expressed in the developing hypothalamus [41, 52].

**Table 3:** Comparison of proneural gene expression in the chick and mouse brains

	*Ascl1*		*Neurog1*		*Neurog2*	
	Chick	Mouse	Chick	Mouse	Chick	Mouse
nmesV	✓	✓	✓	✓	✓	
nMLF			✓	✓	✓	✓
nMTT			✓	✓	✓	✓
nTPC	✓	✓				
nTPOC	✓	✓				
nIII			✓	✓	✓	✓
nIV	✓	✓	✓			

Ticks indicate where expression was located in early axon scaffold neuronal populations and motor neurons at HH18 in chick and E10.5 in mouse

*Ascl1* was first expressed in the brain from E8 along the dorsal midline of the mesencephalon [56]. *Neurog1* was also first expressed along the dorsal midline of the mesencephalon, slightly later at E8.5 (Fig. 3b). This expression of *Ascl1* (Fig. 3a) and *Neurog1* corresponded to the positioning of the nmesV. *Neurog2* was first expressed at E8.5 in the ventral brain, corresponding to the nMLF (Fig. 3c).

By E9.5, while *Ascl1* expression was mostly restricted to the dorsal midline of the mesencephalon (Fig. 3d), *Neurog1* expression expanded throughout the entire mesencephalon (Fig. 3e) and *Neurog2* was not expressed in the dorsal mesencephalon (Fig. 3f). At this stage, *Ascl1* was also expressed in the nTPOC, nTPC and Pth (Fig. 3d), *Neurog1* was expressed in the nMLF (Fig. 3e) and *Neurog2* was expressed in the nMTT, nMLF, the caudal thalamus (Fig. 3f; unfilled arrowhead) and in the dorsal optic vesicle (Fig. 3f; arrowhead).

At E10.5, *Ascl1*, *Neurog1* and *Neurog2* were differentially expressed throughout the early neuronal populations of the developing brain (Fig. 3g, h, i, j and Table 2). For example, both *Neurog1* and *Neurog2* were expressed in the caudal thalamus (Fig. 3h, i, unfilled arrowhead), the nMLF and the nIII (Fig. 3h, i), while *Ascl1* expression was restricted either side of the caudal thalamus in the Pth and in the Ptec (Fig. 3g). By E10.5, the mesencephalon contained both DTmesV neurons along the dorsal midline and cda neurons that were not clearly distinct from each other [33]. Expression of *Neurog1* overlapped with both the cda and nmesV (Fig. 3h), while *Ascl1* expression was more nmesV specific (Fig. 3g).

In the prosencephalon and mesencephalon, there was very little overlap between the expression of *Ascl1* and the two Neurogenin genes. The only exception was at the level of the nmesV (Fig. 3g, h, i; Table 3) where *Ascl1* and *Neurog1* expression overlapped. This mutual exclusivity of proneur﻿al gene expression was especially obvious at the level of the nTPC and the cortex (Fig. 3g, h, i). With respect to the neuronal populations of the early axon scaffold tracts, the nTPC and nTPOC were the only populations to express a single proneural gene, *Ascl1* (Fig. 3g). Although the nTPOC only expressed *Ascl1* here, *Neurog3* was also expressed in the hypothalamus, although not in this specific set of the early neurons [52, 53].

These expression studies have revealed a close relationship between proneural and neuronal markers in the developing mouse brain. In order to test whether the neuronal markers described in this study were specific targets of these proneural genes we decided to use the chick model. Therefore, we needed to determine whether expression of the proneural genes was conserved in the early neuronal populations by analysing and comparing the expression patterns of *Ascl1*, *Neurog1* and *Neurog2* in the developing chick brain.

### Differential expression of proneural genes was highly conserved between the chick and mouse brains

**Fig. 4:**
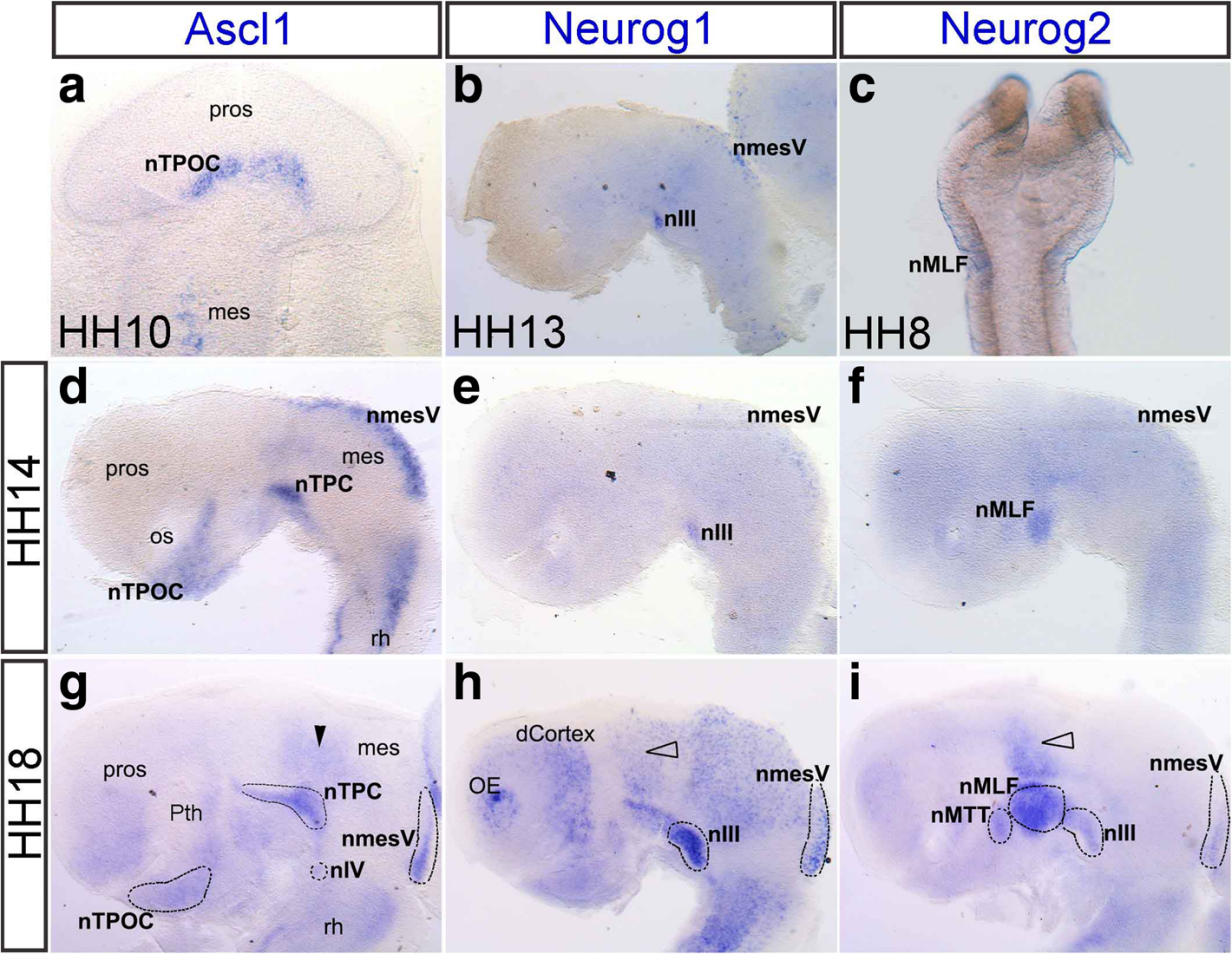
*Ascl1*, *Neurog1* and *Neurog2* expression in complementary regions of the chick brain. **a**-**c** First expression of *Ascl1* (**a**, ventral view) at HH10 in the hypothalamus, *Neurog1* (**b**, dissected, lateral view) at HH13 in the mesencephalon and *Neurog2* (**c**, ventral view) at HH8 in the nMLF. **d**-**f** HH14 (dissected brain, lateral view). Expression of *Ascl1* (**d**), *Neurog1* (**e**) and *Neurog2* (**f**). **g**-**i** HH18 (dissected brain lateral view). Expression of *Ascl1* (**g**), *Neurog1* (**h**) and *Neurog2* (**i**). Expression in the pretectum (arrowhead). Expression in the caudal thalamus (unfilled arrowhead). For abbreviations see Table 1

In the developing chick brain, *Neurog2* was the first proneural gene to be expressed from HH8 in the progenitors that will give rise to the MLF neurons (Fig. 4c). *Ascl1* was first expressed in the brain at HH10 corresponding to the nTPOC (Fig. 4a). The expression of these proneural genes predated any of the downstream target genes and differentiated neuronal populations [44, 57]. *Neurog1* was first expressed in the brain from HH13 within the nmesV and nIII (Fig. 4b). Expression of *Ascl1* expanded to the nmesV from HH11 (data not shown), and then at HH14 the nTPC (Fig. 4d). By HH18, expression of *Ascl1* (Fig. 4g), *Neurog1* (Fig. 4h) and *Neurog2* (Fig. 4i) was in various neuronal populations of the early axon scaffold tracts and the motor neurons. *Neurog2* was expressed in the nMTT and dorsally above the MLF (Fig. 4h, arrowhead). Similar to mouse, the expression of these genes was mostly in complementary populations, expression of all three proneural genes only overlapped in the dorsal mesencephalon within the nmesV (Fig. 4g, h, i). *Neurog1* and *Neurog2* also overlapped in the nIII (Fig. 4h, i). From HH18, proneural genes were expressed in other neuronal populations of the brain. For example, expression of neurogenins dorsal to the MLF in both chick and mouse corresponded to the caudal thalamus (Fig. 4g, h. i, unfilled arrowhead).

We showed that the expression of these proneural genes in the chick and mouse brains was highly conserved, however, there were some slight differences (Table 3). For example, *Neurog2* was expressed in the chick nmesV (Fig. 4i), but not in the mouse (Fig. 3i). Compared with mouse, there was less overlap of all the proneural genes in the chick as *Neurog2* was not as widely expressed throughout the populations in chick (Table 3). Interestingly, while the expression domains were conserved, the timing of expression was not always the same. For example, *Neurog2* expression was switched on first in chick (Fig. 4c), while *Ascl1* expression was switched on first in mouse. This was likely to be a reflection of the difference in timing of the first neuronal populations forming in the brain. The nmesV formed first in mouse [12] and the nMLF formed first in chick [57].

### Expression of proneural genes overlapped with the expression of neuronal markers in the early neuronal populations of both the chick and mouse brains

Together, the proneural genes analysed here overlapped with the expression of all the neuronal markers in both the chick and mouse (Figs. 1, 3, 4). However, their expression did not correlate completely with either the domain of *Ascl1* or the neurogenins. In terms of neuronal marker expression, no single proneural gene completely overlapped with the complete expression of a target gene. *Tagln3* expression, for example, did not completely overlap with *Ascl1* (Figs. 1l and 3g). In chick, *Tagln3* expression was detected in the nMLF and *Neurog2* was the only proneural gene to be expressed in this region, while in mouse both *Neurog1* and *Neurog2* were expressed. This expression analysis suggested that different proneural genes were likely to regulate the same neuronal markers. In contrast to this observation, in both chick and mouse, *Chga* was specifically expressed in the nTPC with *Ascl1* being the only proneural gene in this population (Figs. 1m, 3g, 4g). To test this specificity, we overexpressed *Ascl1* and *Neurog2* in the chick brain.

### *Ascl1* overexpression induced ectopic neuronal differentiation and misguided axon projection in the developing chick mesencephalon

**Fig. 5:**
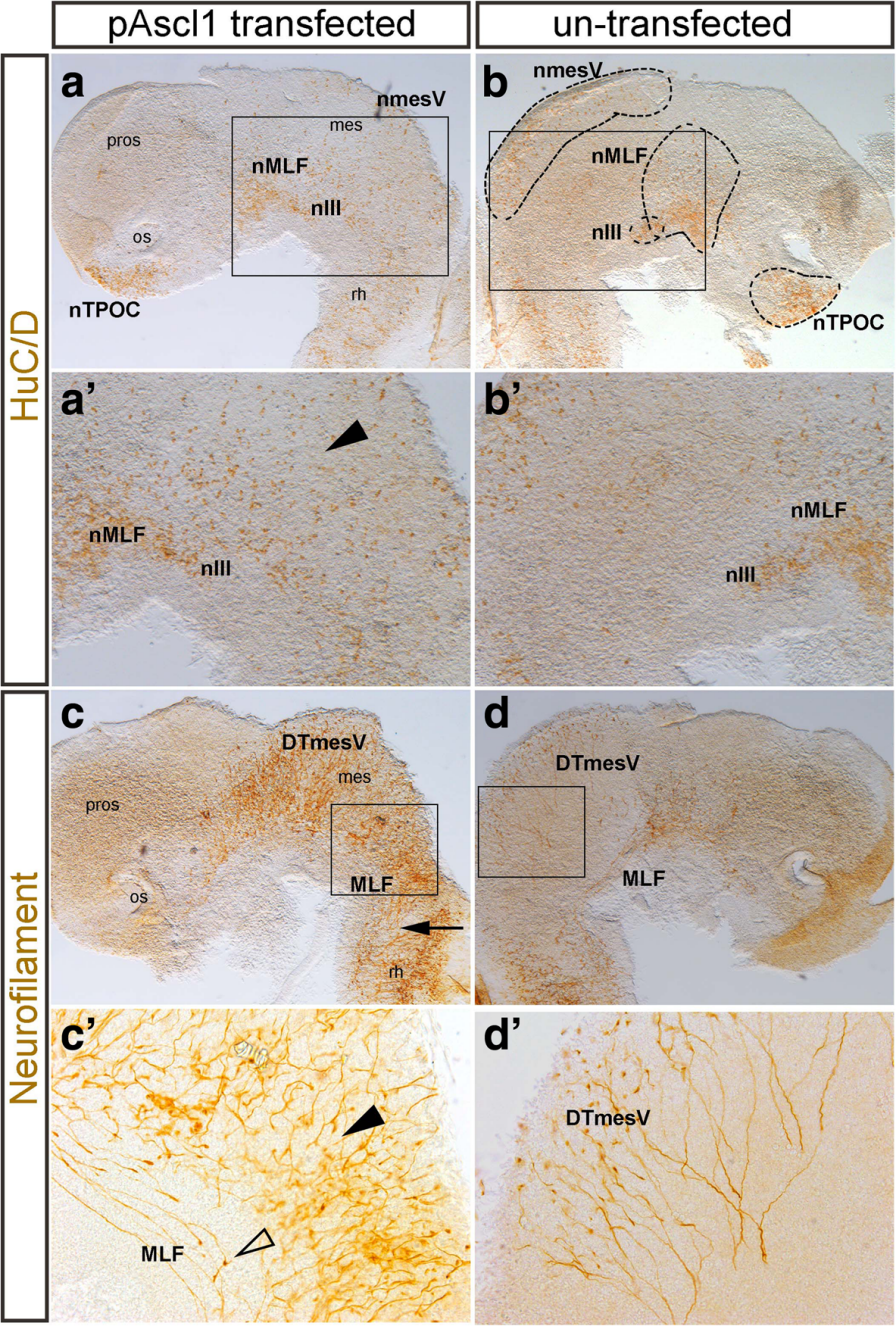
*Ascl1* overexpression leads to ectopic neuronal differentiation. All brains have been dissected, flatmounted and in lateral view. **a**, **b**, *a’*, *b’*; *n* = 3 The neuronal populations were labelled with HuC/D in the chick brain after electroporation with the pAscl1 plasmids. Box indicates higher magnification image. **a**, *a*’ More HuC/D positive cells were visible in the mesencephalon (arrowhead). **b**, *b*’ The un-transfected half of the brain showed normal distribution of neurons. **c**, **d**, *c’*, *d’*; *n* = 3 The neuronal populations and their associated axon tracts were labelled with Neurofilament in the chick brain after electroporation with the pAscl1 plasmid. **c** There was an increase in the number of neurons and axons in the mesencephalon. Some of these neurons projected axons into the hindbrain (arrow), not seen in control side (**d**). Box indicates higher magnification image. (*c’*) Some axons did not project correctly. In the ventral brain axons projected rostrally towards the DMB (arrowhead) and other axons within the mesencephalon projected in a curved shape (arrowhead), not directly ventral like the axons in the control (**d**). **d**, *d’* Normal distribution of neurons and axons projected in the correct way. For abbreviations see Table 1

Previously, upregulation of *Ascl1* in other regions of the embryo led to increased number of neurons [4, 15, 24]. First, the identity of the cells that were electroporated and subsequently overexpressed *Ascl1* was investigated using HuC/D and Neurofilament pan-neuronal antibodies. Embryos were electroporated at HH10, just after neural tube closure, targeting the mesencephalic cells as the proneural and neuronal markers were not widely expressed in this region and there were few post-mitotic neurons (Fig. 5b, d). After 24 h, the number of HuC/D positive post-mitotic neurons increased when *Ascl1* was overexpressed in the chick brain (Fig. 5a, *a*’ arrowhead; *n* = 3). These results confirmed that the *Ascl1* construct used here had the ability to induce neurogenesis in cells that were not yet destined to become neurons. Eventually neurons in this region will become tectobular forming the ventral commissure [57]. While HuC/D only showed an increase in the number of neurons, Neurofilament labelled both neurons and their projecting axons (Fig. 5c, d). Interestingly, some of these axons appeared to project along the same path as the DTmesV axons into the rhombencephalon (Fig. 5c, arrow). However, some axons were projecting rostrally back towards the diencephalic-mesencephalic boundary (DMB) (Fig. 5*c’*, unfilled arrowhead), and some axons appeared to be curling back on themselves (Fig. 5*c’*, arrowhead). These results confirmed neurons differentiated from cells that ectopically expressed *Ascl1*, however, their ability to follow the correct path was affected.

### Overexpression of *Ascl1* and *Neurog2* caused ectopic expression of the same target genes in the chick brain

To establish a possible specificity of the proneural gene for one of the neuronal markers, we electroporated *Ascl1* and *Neurog2* and analysed the effect on expression of the neuronal markers *Nhlh1*, *Tagln3*, *Chga* and *Stmn2*.

**Fig. 6:**
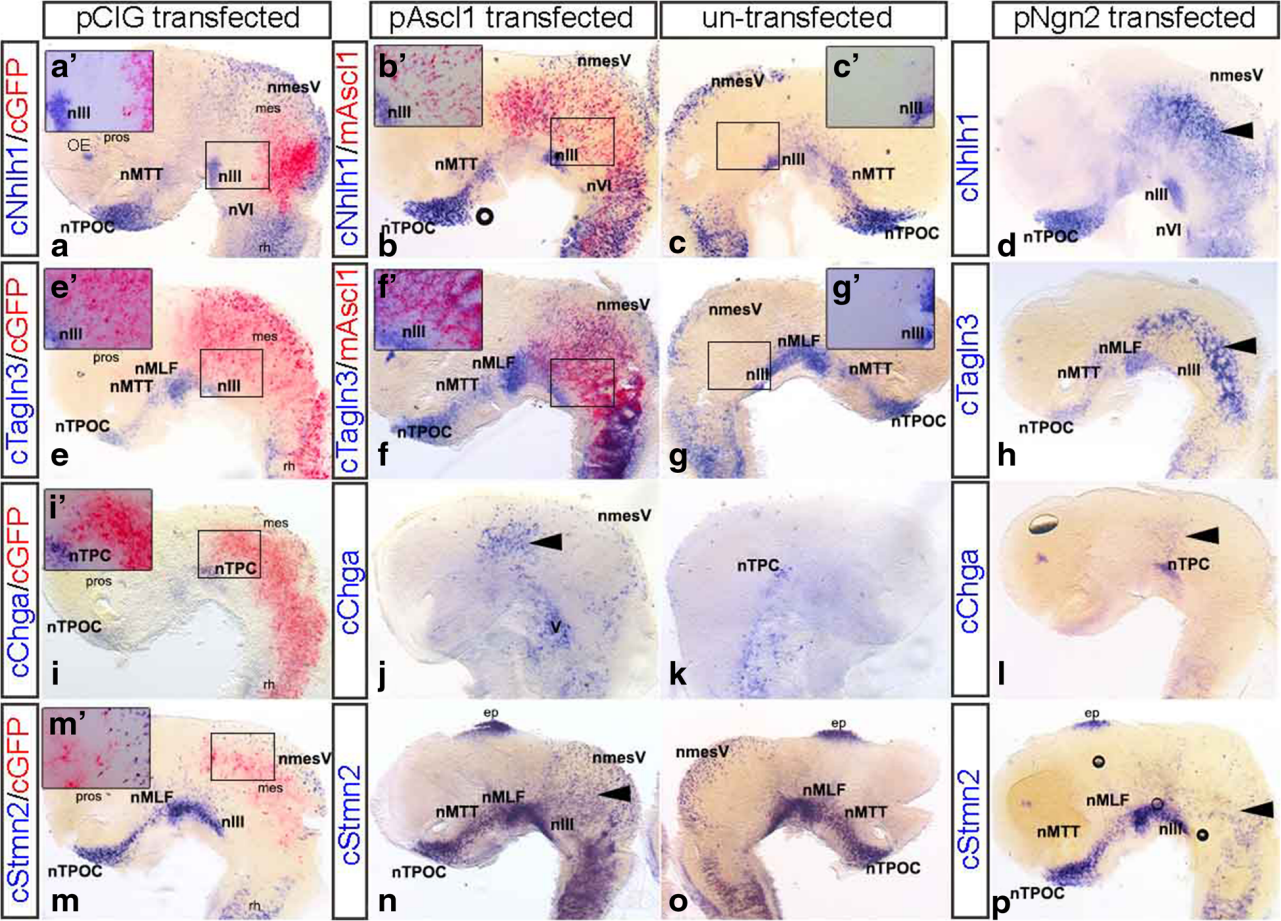
Overexpression of *Ascl1* and *Neurog2* caused upregulation of *Nhlh1*, *Tagln3, Chga* and *Stmn2.* **a**-**p**, minimum *n* = 3 for each gene) All brains have been dissected, flatmounted and in lateral view. **a**, a’, **e**, *e’*, **i**, *l*’, **m**, *m*’ Normal expression of *Nhlh1*, *Tagln3, Chga* and *Stmn2* within neurons of the early axon scaffold in the control embryos, with GFP (red) specifically labelling cells that express the control plasmid (pCIG; CAGGS-IRES-nuclearGFP). Expression of *Nhlh1* (**b**, *b*’), *Tagln3* (**f**, *f*’)*, Chga* (**j**; arrow) and *Stmn2* (**n**; arrow) was upregulated in cells where r*Ascl1*-IRES-nuclearGFP was ectopically expressed. (**b**, *b*’, **f**, *f*’) *mAscl1* can be specifically labelled (red) to show co-expression with the target genes *Nhlh1* and *Tagln3*. (**c**, *c’* **g**, *g’* **k**, **o**) Normal expression was also observed on the un-transfected (internal control) side of the same electroporated embryo. Ectopic expression of m*Ngn2*-IRES-nuclearGFP also resulted in ectopic expression of *Nhlh1* (**d**)*, Tagln3* (**h**)*, Chga* (**l**) and *Stmn2* (**p**). The un-transfected (internal control) was not displayed here. For abbreviations see Table 1

In embryos electroporated with the pCIG control plasmid (n ≥ 3), no ectopic expression of *Nhlh1*, *Tagln3*, *Chga* and *Stmn2* was observed in cells expressing the control plasmid and each gene was normally expressed within the early neuronal populations (Fig. 6a, e, i, m). When either rat *Ascl1* (minimum *n* = 3 for each gene) or mouse *Neurog2* (minimum *n* = 3 for each gene) were overexpressed, cells that ectopically expressed the proneural gene, also expressed the markers *Nhlh1* (Fig. 6b, d), *Tagln3* (Fig. 6f, h), *Chga* (Fig. 6j, l) and *Stmn2* (Fig. 6n, p). As rat and mouse sequences were used, the ectopically expressing cells could be labelled specifically with a rat or mouse RNA riboprobe, therefore highlighting only the cells that were ectopically expressing the gene (Fig. 6; red). As only one half of the brain was electroporated, the other half acted as an internal control (Fig. 6c, g, k, o). The un-transfected side of the embryo showed no ectopic expression of the gene and resembled the pCIG embryo. *Pax6* and *Sox10* were tested as negative controls to confirm the specificity of the electroporation, as they were not known to be downstream targets of proneural genes. When *Ascl1* was overexpressed, neither *Pax6* (Additional file 1: Figure S1A, B; *n* = 3) or *Sox10* (data not shown; *n* = 3) were upregulated. Together, these results suggested that both ASCL1 and NEUROG2 were able to regulate the same neuron specific genes tested here.

### Loss of *Ascl1* led to discrete loss of *Tagln3* and *Chga* expression in the developing mouse brain

**Fig. 7:**
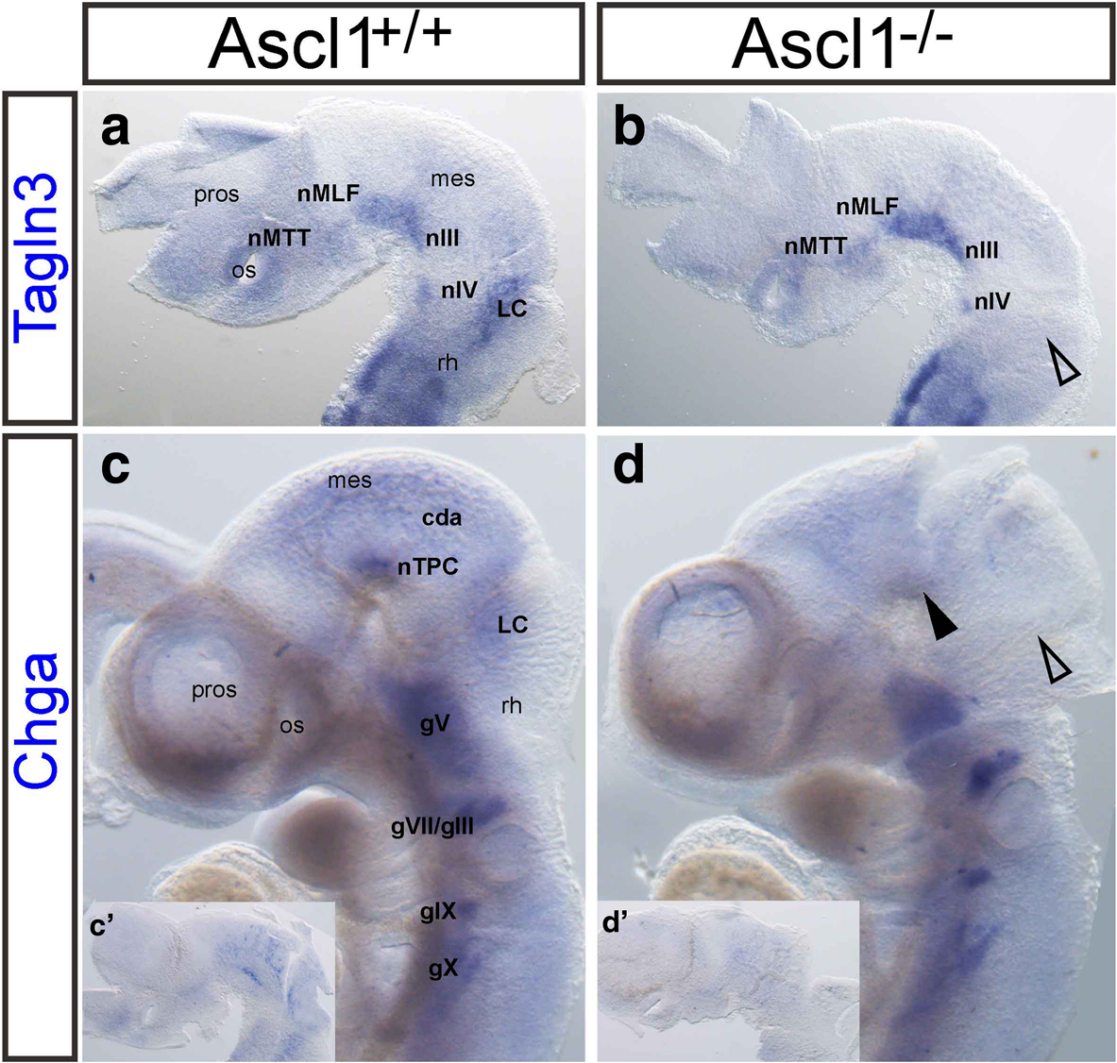
Loss of *Ascl1* led to very specific downregulation of *Tagln3* and *Chga.* Expression of *Tagln3* in control (**a**, *n* = 3) and *Ascl1* null mutant embryos (**b**, *n* = 3). Expression was lost specifically in the locus coeruleus (LC; unfilled arrowhead). (**c**, **d**) Whole mount embryos. Expression of *Chga* in control (**c**, *n* = 3) and Ascl1 null mutant embryos (**d**, *n* = 2). Expression was specifically lost in the nTPC (filled arrowhead), LC and cda. (*c’*, *d’*) Inserts indicate *Chga* expression in flatmounted brains in lateral view of the embryos in **c** and **d**. For abbreviations see Table 1. gV: trigeminal ganglion; gVII/VIII: facial and vestibulocochlear ganglia; gIX: petrosal ganglion; gX: nodose ganglion

*Ascl1* was specifically expressed in some neuronal populations where other proneural gene expression was missing, for example, in the nTPC (Fig. 3g). Therefore, to determine whether *Ascl1* had a specific role in the regulation of the neuronal genes within the early neuronal populations, *Ascl1* null mutant embryos were analysed to investigate the expression of the pan-neuronal gene *Tagln3* (Fig. 7; *n* = 3). Surprisingly, *Ascl1* null mutant embryos still expressed *Tagln3* in all of the neuronal populations at E10 (Fig. 7b), except the LC (Fig. 7b, unfilled arrowhead). The LC was already known to be affected in *Ascl1* mutant mice [22, 37]. We also investigated the expression of *Chga* in *Ascl1* null mutant embryos as its expression was more specific in the early neuronal populations (Fig. 1). Remarkably, in the *Ascl1* mutant embryos, *Chga* expression was specifically lost in the nTPC, while expression in the ganglia was not affected (Fig. 7d, d’, filled arrowhead; *n* = 2). *Chga* expression was also downregulated in the cda and in the LC (Fig. 7d, unfilled arrowhead) compared with the control embryos.

## Discussion

The organisation of the initial neuronal populations of the brain giving rise to the early axon scaffold has been studied in great detail in zebrafish, chick and mouse [33, 57, 59]. However, the molecular mechanisms that underlie the specification of these early differentiating neurons remain undetermined. Our study shows that differentiation of these neurons is tightly regulated by the Notch/proneural network and reveals important new expression descriptions of proneural and neuronal markers in the early axon scaffold in both chick and mouse. This work adds further evidence to suggest evolutionary conservation of the genetic mechanisms that control neuron differentiation between birds and mammals.

### Expression of specific neuronal markers reveals genes that potentially play an essential role in the differentiation and specification of the populations that give rise to the early axon scaffold

Very few specific markers are described in the individual neuronal populations of the developing vertebrate brain at early stages during the formation of the early axon scaffold tracts. This study describes 5 genes, *Nhlh1*, *Tagln3*, *Chga, Cntn2* and *Stmn2* that are expressed in specific neuronal populations and play a role in the Notch/proneural network. These are all known neuronal markers that mediate critical biological processes required to induce neuronal identity [35, 44]. *Nhlh1* and *Tagln3* are involved in fate determination, whereas *Chga*, *Cntn2* and *Stmn2* are expressed during terminal differentiation. There is some evidence that these neuronal genes play a specific role in determining the identity or function of these distinct neuronal clusters. For example, *Cntn2* has a role in the guidance of the MLF axons [61], and the specific expression of *Chga* in the nTPC in both the chick and mouse bra﻿ins﻿, suggests that nTPC may have a neuroendocrine function [49].

Despite the fact that *Nlhh1*, *Tagln3,* and *Stmn2* are considered pan-neuronal markers they have, to some extent, specific expression at the level of the first neurons establishing the early axon scaffold tracts in the amniote brain [55]. We show that each of these neuronal populations have a specific combination of these neurogenic markers during differentiation (Table 2). This means that very early during development these neurons acquire a specific identity. Most importantly, with a few exceptions, the expression pattern of these neuronal markers is highly conserved between chick and mouse (Table 2). Still, it is surprising to see that *Nhlh1* and *Tagln3* are not expressed in the mouse nmesV until after the first neurons differentiated at E8.5 [55], whereas *Nhlh1* and *Tagln3* are early markers for post-mitotic neurons in the chick [44]. Further analysis will be required to determine the function of this discrepancy as ultimately these neuronal populations express the same genes in both the chick and mouse brains.

### A relationship between Notch signalling, proneural genes and downstream targets is essential for the correct patterning of early neuronal populations in the developing vertebrate brain

Numerous studies support the idea that the Notch signalling pathway and proneural genes act together in a feedback loop to promote initial neurogenesis [5, 10, 29, 43]. However, in the developing brain, this has only been observed in the chick embryo via DAPT treatment [43]. By the inhibition of Notch signalling, this study confirms the role of Notch signalling in the Notch/proneural molecular circuitry that operates within the developing mouse brain similar to the other neural structures to control neurogenesis.

### Compensation by proneural genes is not neuronal population specific

We show that a complex pattern of proneural gene expression exists during the generation of the initial neuronal populations in the brain. This seems to be the general situation in most regions of the central nervous system [32]. Therefore, it is not surprising that *Ascl1* and *Neurog1/2* play a central role in the selection of neuronal progenitor subtypes by regulating downstream target genes [2, 5, 13, 37]. Genomic approaches (CHIP on chip, ChIP-seq and RNA-seq) are powerful tools that have led to the identification of hundreds of targets of ASCL1 [6, 8] and NEUROG2 [28]. However, the relationship between the proneural genes and these target genes, is yet to be functionally shown. In the present study, as the neuronal markers *Nhlh1*, *Tagln3*, *Chga, Cntn2* and *Stmn2,* are expressed in very similar expression patterns to the proneural genes, we propose that precise proneural genes regulate expression of specific neuronal genes, including, in specific neuronal populations of the early axon scaffold tracts.

Interestingly, we show that the nTPC has a very specific expression identity. These neurons do not express the pan-neuronal markers *Nhlh1* and *Talgn3*, they are the only neurons to have a strong expression of *Chga*, and *Ascl1* is the exclusively expressed proneural gene. Furthermore, in both the chick and mouse brains, expression of *Chga* is excluded from neuronal populations expressing *Neurog1* and *Neurog2.* This observation strengthens the argument for a specific function of ASCL1 in the development of specific neuroendocrine neurons [34], and this is in accordance with the downregulation of *Chga* in the *Ascl1* null mutant embryo.

This study shows that regulating expression of the target genes analysed here is not specific to either the overexpression of *Ascl1* or *Neurog2*, suggesting proneural genes are functionally equivalent (at least to induce neuronal identity). Indeed, while proneural genes are expressed in complementary regions, there are numerous studies that show they able to compensate for each other [26, 37, 45]. It has been demonstrated that *Neurog2* has the capacity to rescue the development of *Ascl1*-dependent neurons [34, 37]. It is therefore not surprising that in the *Ascl1* null mutant embryos, the expression of *Tagln3* is not downregulated in neuronal populations expressing more than one proneural gene. This suggests there is compensation of other proneural genes in these populations. However, *Tagln3* expression is not downregulated in the nVI where *Ascl1* is the exclusively expressed proneural gene is unexpected. Other known proneural genes, *Neurog1*, *Neurog2*, *Neurog3* and *Atoh1* seem to be not expressed in the nVI. What is regulating *Tagln3* here is yet to be determined.

The highly conserved expression patterns of the proneural genes in the early ventral forebrain argue against a model of stochastic induction. An important selection pressure may exist to maintain this complementary proneural gene expression within the chick and mouse brains. We still have to determine why these neuronal target genes are expressed in some populations but not others, especially if these genes can be regulated by any proneural gene. It has been demonstrated, that these proneural genes are not always functionally equivalent and this capacity appears to vary in different regions of the nervous system [37]. How the divergent function of the proneural genes is established remain ambiguous. Further analysis of mice containing targeted mutations in both the *Ascl1* and *Neurog2* genes should be informative in answering this question.

### The regional cues are likely to be involved in controlling the position of the various neuronal populations that give rise to the early axon scaffold tracts

Questions still remain, including what is controlling the specification of the individual neuronal populations that give rise to the early axon scaffold tracts and other early populations.

If proneural genes can regulate the same target genes, we still need to determine the specific genes or combination of genes (in a cascade) that regulate identity of each individual neuronal population of the early axon scaffold. Although a single proneural gene is sufficient to induce neuronal features, the additional expression of other factors is necessary to generate specific identity, for example, in fibroblasts [51, 54]. Thus, there is another layer of complexity with other regional cues such as those produced by homeobox genes [17, 42]. Specification of neurons in the neural tube relies on combinations of bHLH and other transcription factors to activate or repress specific neurogenic programs. Homeobox genes, such as, *Sax1* could play a role in specifying the nMLF subtypes [46], as gain of function of *Sax1* results in an enlargement of the nMLF area [1]. However, other homeobox genes need to be found in order to explain the patterning of the neuronal populations of the early axon scaffold tracts.

Initially, a critical step is the establishment of morphogen gradients controlling the distinct sets of transcription factors resulting in the establishment of progenitor domains [25]. Such a mechanism has not yet been described during the establishment of the progenitor domains of the axon scaffold. It may be a different mechanism, as these populations of neurons are not distributed along specific axis. Sonic hedgehog (SHH), one of the main signalling molecules involved in neurogenesis patterning [38] is differentially expressed in the ventral forebrain [56] and mostly likely plays a critical role in the formation of the early axon scaffold tracts [1].

## Conclusions

The organisation of the brain is more complex and harbours a greater diversity of neurons compared with the spinal cord. However, to our knowledge, no study investigating the specification of the neuronal populations that give rise to the early axon scaffold in any mutant mouse models has been done. Our present study gives essential tools to explore more accurately the formation of these neuronal populations in mutant models. This will provide a better understanding of how these early neurons differentiate in a specific territory with a specific identity.

## Additional file

Additional file 1: Figure S1.
*Ascl1* overexpression did not cause ectopic expression of *Pax6*. (**a, b**, *n* = 3) Brain was dissected, flatmounted and in lateral view. There was no upregulation of *Pax6* when the embryo was electroporated with the pAscl1 plasmid, which confirmed the specificity of the plasmid. The un-transfected side also showed normal expression of *Pax6*. For abbreviations see Table 1. (PDF 486 kb)
