# Supplementary material for: Regulation of downstream neuronal genes by proneural transcription factors during initial neurogenesis in the vertebrate brain
Source: Neural Dev. 2016 Dec 7;11:22. doi: 10.1186/s13064-016-0077-7 (PMC5142277; doi:10.1186/s13064-016-0077-7)
Supplement: Additional file 1: Figure S1. — Ascl1 overexpression did not cause ectopic expression of Pax6. (a, b, n = 3) Brain was dissected, flatmounted and in lateral view. There was no upregulation of Pax6 when the embryo was electroporated with the pAscl1 plasmid, which confirmed the specificity of the plasmid. The un-transfected side also showed normal expression of Pax6. For abbreviations see Table 1. (PDF 486 kb) [file 13064_2016_77_MOESM1_ESM.pdf]

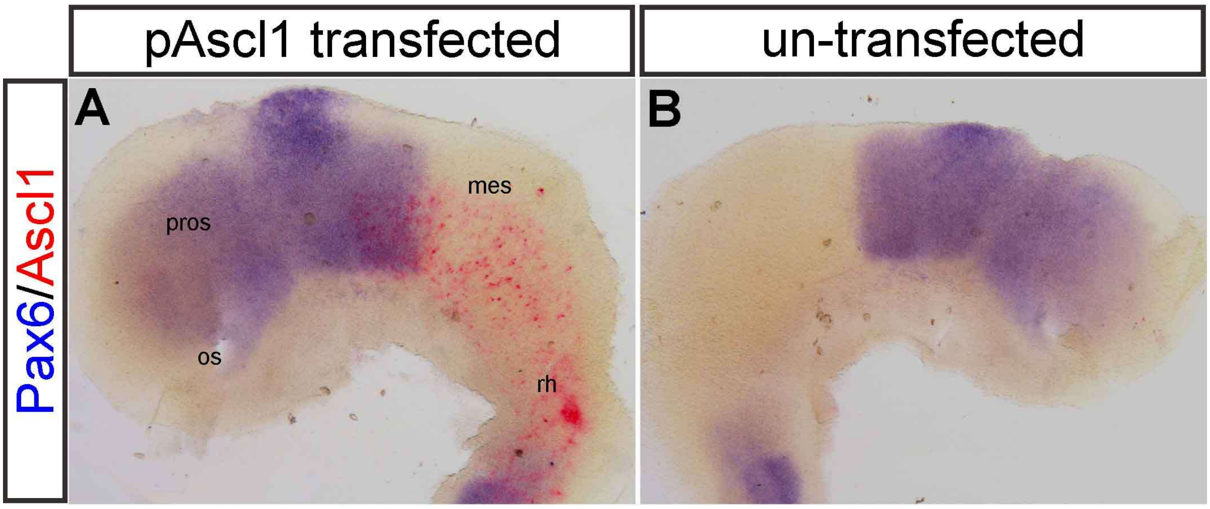

Supplementary Figure 1. *Ascl1* overexpression did not cause ectopic expression of *Pax6*

(A, B, n=3) Brain was dissected, flatmounted and in lateral view. There was no upregulation of *Pax6* when the embryo was electroporated with the pAscl1 plasmid, which confirmed the specificity of the plasmid. The un-transfected side also showed normal expression of *Pax6*. For abbreviations see Table 1.
